# Targeted Intracellular Delivery of Antibodies: The State of the Art

**DOI:** 10.3389/fphar.2018.01208

**Published:** 2018-10-24

**Authors:** Tatiana A. Slastnikova, A. V. Ulasov, A. A. Rosenkranz, A. S. Sobolev

**Affiliations:** ^1^Laboratory of Molecular Genetics of Intracellular Transport, Institute of Gene Biology, Russian Academy of Sciences, Moscow, Russia; ^2^Faculty of Biology, M. V. Lomonosov Moscow State University, Moscow, Russia

**Keywords:** antibody intracellular delivery, subcellular drug delivery, protein delivery systems, cancer, intracellular transport

## Abstract

A dominant area of antibody research is the extension of the use of this mighty experimental and therapeutic tool for the specific detection of molecules for diagnostics, visualization, and activity blocking. Despite the ability to raise antibodies against different proteins, numerous applications of antibodies in basic research fields, clinical practice, and biotechnology are restricted to permeabilized cells or extracellular antigens, such as membrane or secreted proteins. With the exception of small groups of autoantibodies, natural antibodies to intracellular targets cannot be used within living cells. This excludes the scope of a major class of intracellular targets, including some infamous cancer-associated molecules. Some of these targets are still not druggable via small molecules because of large flat contact areas and the absence of deep hydrophobic pockets in which small molecules can insert and perturb their activity. Thus, the development of technologies for the targeted intracellular delivery of antibodies, their fragments, or antibody-like molecules is extremely important. Various strategies for intracellular targeting of antibodies via protein-transduction domains or their mimics, liposomes, polymer vesicles, and viral envelopes, are reviewed in this article. The pitfalls, challenges, and perspectives of these technologies are discussed.

## Introduction

First established in the 1950s, the drug delivery field has since grown substantially and evolved through several generations (Yun et al., 2015), from simple formulations for oral and transdermal delivery to complex delivery systems with the ability to overcome biological barriers. A simple search for “drug delivery” in PubMed reveals a slow growth in the number of results until the early 2000s, followed by rapid expansion up to 2015, and a subsequent plateau of approximately 15,000 publications per year. Nevertheless, a tremendous number of in-demand drugs exert effects within cells, and an increasing number of new drugs intended to influence protein regulatory pathways (Higueruelo et al., 2013) are realized through protein–protein interactions (PPI). Pharmaceuticals acting on intracellular macromolecules must be delivered intracellularly into the target cells. Small molecules, which include most drugs, often cross biological membranes readily owing to their size, amphiphilic nature, and the availability of transmembrane carriers. Meanwhile, despite the generally successful transmembrane penetration of many small molecules, some potential intracellular targets are excluded from their modulation. Among such elusive targets, are certain PPIs that regulate essential cellular functions and contribute to the signaling pathways involved in cancer pathogenesis (Lazo and Sharlow, 2016). Small molecules are often less effective than macromolecules for the interruption of PPIs, except for proteins with small-molecule-binding pockets such as enzymes, ion channels, and cell receptors (Crews, 2010), because of the large flat contact areas and lack of poorly defined deep pockets and grooves in which small molecules could insert and subsequently perturb its activity. PPIs often involve large surfaces, which typically range from 1500 to 3000 Å^2^, compared with 300–1000 Å^2^ in protein interactions with small molecules (Wells and McClendon, 2007). A typical example of PPIs is the interaction of antibodies with protein antigens. In recent years, trends have revealed the growing impact of approved polypeptide macromolecular drugs, particular antibodies to cell-surface and soluble targets (de la Torre and Albericio, 2018; Strohl, 2018).

The size of the human interactome size was estimated to include 650,000 or more distinct interactions (Stumpf et al., 2008), but this estimation has since increased (Kotlyar et al., 2017). Recent analysis has revealed that only a small part of the human proteome (less than 700 proteins) is pharmaceutically accessible by approved drugs (Santos et al., 2017), which has led to the concept of “undruggable” targets that are not amenable to small-molecule interventions. This list includes certain infamous and notorious cancer-causing molecules, such as c-Myc, Ras, and NF-κB (Cox et al., 2014; Lazo and Sharlow, 2016; Dang et al., 2017). Intracellular proteins that act at immune checkpoints are other possible targets (Klepsch et al., 2018) for which inhibition by antibodies might reactivate the host’s immune response against cancer cells. In this regard, a huge field of applications has been opened for intracellular treatment and diagnostics using antibodies. One mechanism of antibody action is the alteration of signal transduction through a physical block of the interaction between two proteins that are components of a cellular pathway (Adams and Weiner, 2005). This is exemplified by antibodies binding receptors, such as those of the EGFR family, on cell membranes, and sterically preventing them from transmitting a signal (Weiner et al., 2010). The tumor signaling perturbation through antibody interference has been shown to be a successful approach for the treatment of some diseases (Lenz, 2006; Hudis, 2007). The feasibility of antibodies to downregulate intracellular targets has been proven in experiments with microinjections (Riabowol et al., 1988; Gire and Wynford-Thomas, 1998; Ma et al., 1999) and transfections with a corresponding gene (intrabody approach Southwell et al., 2009; Ryan et al., 2010; Amici et al., 2016). However, the microinjection technique is limited to a small number of cells *in vitro*. The intrabody format has the potential for development, but still exhibits the same risks as gene therapy: the possible genome integration of viral vectors, low efficiency, and possible toxicity of non-viral vehicles, which has necessitated the development of direct antibody introduction methods. Currently, the routine introduction of antibodies into cells to visualize the cellular targets requires membrane permeabilization, which prevents the practical implementation of antibodies against intracellular targets for therapeutic purposes. Thus, the development of convenient technologies for the targeted delivery of antibodies, their fragments, or antibody-like molecules inside living cells without harming the cells is urgently needed. This overall task contains several stages, such as target-cell recognition, internalization, and transport to a relevant intracellular compartment. To date, the relevant literature details different approaches to the delivery of antibodies to their intracellular targets: transfection, cell-penetrating peptides, fragments of bacterial toxins, lipid-based molecules, physical methods (electroporation and microinjection). None of these methods have provided a complete solution for all the necessary stages of intracellular transport. In this review, we have analyzed different approaches intended for recombinant antibody delivery and discussed their limitations and pitfalls.

## Antibodies as Prospective Tools for Undruggable Targets

One possible way to engage in the therapy of intracellular “undruggable” protein targets is the use of antibodies. Antibodies could be raised against different protein antigens, including those with flat contact surfaces that are not druggable via small-molecule approaches. Since the first therapeutic antibody entered the market in 1986, various antibodies against different targets have been developed and more than 60 antibody therapeutics have been approved (Carter and Lazar, 2018). The major advantages of antibodies are supreme specificity and affinity for a target, which make them invaluable basic experimental tools, as well as the standard of care, for indications, such as rheumatoid arthritis (Weinblatt et al., 2003; Smolen et al., 2017), psoriasis (Ratner, 2015), solid tumors (Slamon et al., 2011; Larkin et al., 2015; Roviello et al., 2017), and blood cancers (Coleman et al., 2016; Brown et al., 2017). The considerable success of antibody therapy in the clinic has sparked a major effort into antibody research, including the development of engineered humanized molecules with improved physicochemical properties and safety (Beck et al., 2010; Strohl and Strohl, 2012; Weiner, 2015; Elgundi et al., 2017). Similarly, recent large-scale antibody generation projects established a pipeline for the high-throughput development and validation of binder proteins for numerous possible targets (Colwill and Graslund, 2011; Landegren, 2016).

As a potent tool with wide applications, the antibodies can capture splice variants and proteins subjected to post-translational modifications, as well as different protein isoforms and conformational variants. For fundamental purposes, this approach might allow better clarification of the activity of intracellular proteins in their natural environment for better elucidation and validation of disease mechanisms. Furthermore, antibodies that can interact with a particular domain of a target protein are more specific than approaches such as gene knockout, transfection, and changes in gene expression. To date, most antibodies are limited to cell membrane targets (Scott et al., 2012; Carter and Lazar, 2018), such as receptors or secreted proteins, such as cytokines, growth factors, and hormones. The number of transmembrane and secreted proteins is estimated to be approximately 8000–9000 proteins (Miersch and Sidhu, 2016), which implies that two thirds of the proteome could be unlocked for antibody modulation *in vivo*. To this end, the antibody must be delivered inside the living cell.

## Derivatives of Antibodies and Antibody-Like Polypeptides

**FIGURE 1:**
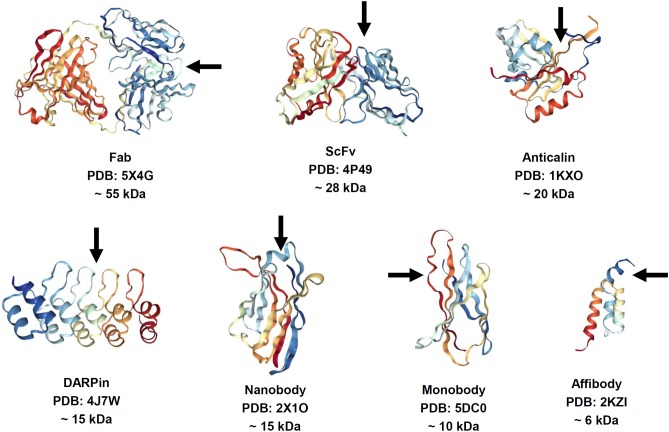
Structure of antibody fragments and alternative antibody scaffolds described in this review. Structures were generated by using NGL Viewer (doi: 10.1093/nar/gkv402). Rainbow color scheme: blue is the N-terminus and red is the C-terminus. Arrows depict antigen binding sites.

The features of antibodies, such as large size, complex architecture and molecular composition, and costly manufacturing, have catalyzed a significant research effort to finding alternative binder molecules based on smaller antibody fragments or alternative protein scaffolds. Thus, 150-kDa multidomain antibodies produced in eukaryotic expression systems have inspired the search for smaller antibody fragments amenable for specific target recognition and suitable for less expensive production in *Escherichia coli* (Figure 1). The most common (Gebauer and Skerra, 2015), 26-kDa ScFv and 45-kDa Fab, are characterized by lower expression costs but also by rapid clearance than the parent molecules. However, they still contain intradomain disulfide bonds, which hamper correct folding upon expression in *E. coli*. Some natural antibodies (for example, those discovered in camels) contain 15-kDa heavy-chain only antibodies lacking a disulfide bond (dsAb or VHH, also known as Nanobodies^®^) (Hamers-Casterman et al., 1993). VHH antibodies are possible alternatives to conventional antibodies, which have good stability, solubility, and expression yields. In addition, dozens of antibody-mimic types have been described through advances in combinatorial selection techniques (Binz et al., 2005; Mintz and Crea, 2013; Skrlec et al., 2015). The most advanced clinical stage scaffolds (Wurch et al., 2012; Gebauer and Skerra, 2015; Vazquez-Lombardi et al., 2015) are a 10-kDa monobody (Adnectin) based on the human fibronectin FN3 domain, a 6-kDa affibody based on the Z-domain of staphylococcal protein A, 20-kDa anticalin derived from human lipocalins, and the 14- to 21-kDa designed-ankyrin-repeat proteins (DARPins) engineered consensus sequence based on ankyrin repeat proteins. Alternative antibody scaffolds are much smaller in size, and combine a disulfide-free single domain with sufficient affinity and easy production in different hosts. A feature of small-size alternative binders and antibody fragments is their short circulation time in the blood, which may be advantageous for diagnostic functionality. Additional opportunities are presented by a class of bispecific antibodies able to recognize simultaneously two different epitopes on the same or different antigens. This concept was proposed for classical immunoglobulins G (IgGs) in 1983 (Milstein and Cuello, 1983) but has been challenging owing to the complexities of their production. Expansion of antibody fragments could push this field forward, which has become a “hot topic” in research since 2010, with more than 60 molecules in development (Del Bano et al., 2015; Godar et al., 2018).

## Internalization of Antibodies Into Cells Via Normal and Pathological Processes

The interior part of a cell is not a single cavity inside the cell plasma membrane, but a sophisticated diversity of compartments separated by intracellular membranes. Therefore, the detection of a substance within a cell can mean quite different things, and the terms “intracellular transport” or “transport into a cell” for an antibody, as well as for any macromolecules, may also have different meanings. In many cases, this transport is limited to entrapment within the compartments of the endocytosis/transcytosis pathway when molecules entering a cell remain within closed membrane formations, from which there were three common pathways: a recirculation path back to the external environment; transcytosis to the other side of the cell; and a degradation path through the lysosomes. However, the most in-demand approach is the transport of antibodies into the cytosol, which may or may not be followed by their subsequent transport to other compartments (e.g., the nucleus), where the majority of potential therapeutic targets are concentrated.

Although it is generally believed that antibodies do not penetrate the plasma membrane, there is a considerable data pool that illustrates the presence of various antibodies within cells (Alarcon-Segovia et al., 1979; Alarcon-Segovia et al., 1996; Reichlin, 1998; Weisbart et al., 1998; Ruiz-Arguelles et al., 2003; Kim J.S. et al., 2015; Weisbart et al., 2015; Noble et al., 2016) as a result of antigen-driven or antibody-driven cell entry of the free antibody or antibody–antigen complex.

Antigen-driven cell entry can be observed for antibodies bound to antigens of several viruses and bacterial toxins that naturally penetrate into cells, mainly through endocytosis followed by subsequent endosomal escape to the cytosol, where they are destroyed by the intracellular Fc-receptor TRIM21 and direct intracellular pathogens into proteasomes (Mallery et al., 2010; Watkinson et al., 2014). TRIM21 is a widely expressed E3 ligase (Yoshimi et al., 2009) recognizing the antibody Fc domain with high affinity (600 pM after TRIM21 dimerization) (Mallery et al., 2010). The function of this system is to protect the cell against intracellular pathogens, such as viruses and bacteria, internalized with attached host antibodies (McEwan et al., 2013). The delivery of an antibody against a particular cell protein could mobilize the TRIM21 system to act against the endogenous proteins (Clift et al., 2017). Nevertheless, to take advantage of the TRIM21 system for the disruption of a particular protein in target cells seems to be a tall order, requiring the delivery of full-sized-antibodies inside living cells.

The intracellular localization of antibodies and their complexes with antigens that enter the cell by antibody-driven mechanisms [e.g., via several types of Fc-receptors (Nimmerjahn and Ravetch, 2008)] is initially normally limited to endocytic vesicles, such as endosomes or phagosomes. Subsequent antibody recycling through neonatal Fc receptors FcRn (Ghetie and Ward, 2002; Ward et al., 2015), and transcytosis or degradation in lysosomes, also prevent antibodies from crossing the membrane and entering the cytosol. However, a recently elucidated mechanism of antigen presentation in dendritic cells, including Sec61-mediated antigen transport from endosomes in the cytosol for cross-presentation (Zehner et al., 2015), might allow the antibody, if already bound to antigen when internalized by the dendritic cell, to enter the cytosol.

As already noted above, the property of most antibodies that limits their applications to targeting extracellular proteins is their inability to cross cellular membranes to reach the cytosol and compartments such as the nucleus. One exception is the small subset of naturally occurring mouse and human autoantibodies that target host self-antigens during autoimmune diseases, such as multiple sclerosis (Douglas et al., 2013), systemic lupus erythematosus, and Sjogren syndrome (Ruiz-Arguelles et al., 2003; Rhodes and Isenberg, 2017) and artificial ones based on them (Jang et al., 2009; Weisbart et al., 2012; Choi et al., 2014). It has been reported that these autoantibodies possess an intrinsic ability to penetrate living cells and capture intracellular and intranuclear antigens, such as DNA, histones, ribosomal protein P, nuclear ribonucleoproteins, and others (Noble et al., 2016). Penetration into different types of cells has been described, but it was not antibody isotype-specific (Rhodes and Isenberg, 2017). In some cases, autoantibody internalization causes cell apoptosis (Sun et al., 2001; Rivadeneyra-Espinoza and Ruiz-Arguelles, 2006; Douglas et al., 2013) or cytokine release (Sun et al., 2000), which contributes to the inflammation associated with autoimmunity. Several mechanisms of internalization have been suggested, including the interactions of basic residues in the complementarity-determining region of autoantibodies with a negatively charged cell surface (Song et al., 2008), Fc receptor-mediated entry (Lisi et al., 2007), or heparin sulfate proteoglycan-mediated clathrin-dependent endocytosis (Choi et al., 2014), or via caveolae/raft-dependent endocytosis (Jang et al., 2009) of nucleoside transporter ENT2 (Hansen et al., 2007). The ability of some autoantibodies to cross the cell membrane was explored with 3D8 and 3E10 anti-DNA autoantibodies as delivery vectors for attached payloads such as nanoparticles (Chen et al., 2016) and proteins (Weisbart et al., 2003, 2004a; Hansen et al., 2006; Hansen et al., 2007). However, a deep understanding of the penetration mechanisms of autoantibody and the extent to which they may be modified is still lacking.

## Strategies for Intracellular Targeting of Antibodies, Their Mimics, and Derivatives

**FIGURE 2:**
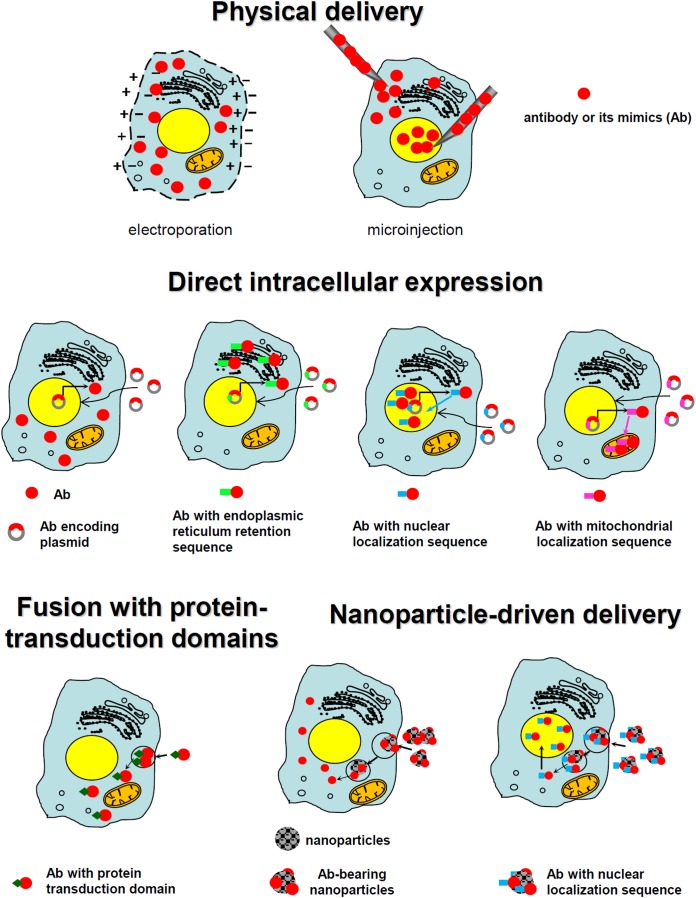
Schematic presentation of the main approaches utilized for the intracellular targeting of antibodies.

Except for the above-described, relatively limited group of antibodies that can penetrate cell membranes, most antibodies targeted to promising intracellular targets do not demonstrate cell-penetrating ability, making their delivery inside a cell a key bottleneck. Various technologies have been developed and tested to accomplish this task; some success has occurred. Those strategies can be grouped into several major classes: direct physical delivery, direct intracellular expression, fusion with the part of internalizing autoantibodies responsible for their intrinsic ability to enter the cells, the use of protein-transduction domains or their mimics, and the use of various nanoparticle carriers (including inorganic nanoparticles, liposomes, polymersomes, and viral envelopes). These main approaches are schematically presented in Figure 2.

### Physical Delivery

One of the most straightforward and oldest strategies of intracellular antibody delivery is their direct physical transfer into the target cell; the predominantly exploited methods include electroporation (Chakrabarti et al., 1989; Lukas et al., 1994; Marrero et al., 1995; Baron et al., 2000; Rui et al., 2002; Freund et al., 2013; Marschall et al., 2014; Desplancq et al., 2016; Conic et al., 2018) and microinjection (Scheer et al., 1984; Riabowol et al., 1988; Lessman et al., 1997; Gire and Wynford-Thomas, 1998; Ma et al., 1999; Keppeke et al., 2015; Dixon et al., 2017). These methods are successfully used in protein functional studies, although a rising number of studies aimed at establishing an *in vivo* electroporation technique (Aihara and Miyazaki, 1998; Lohr et al., 2001; Heller and Heller, 2010), primarily for nucleic acid delivery, including encouraging clinical trials (NCT01440816) (Daud et al., 2008) makes their practical implementation theoretically conceivable for some local applications. Recently, several new physical delivery approaches have been recruited for intracellular antibody delivery. For example, anti-E6 HPV 16 oncoprotein antibody delivered by sonoporation, which is based on the simultaneous use of high intensity focused ultrasound with microbubbles, was shown to restore p53 expression, whose degradation is promoted by the E6 oncoprotein (Togtema et al., 2012). A new sophisticated method called the biophotonic laser-assisted surgery tool, relying on laser-induced cavitation bubbles for the creation of transient plasma membrane pores, was demonstrated to deliver different cargoes, including antibodies, into cells (Wu et al., 2015). In addition, an approach utilizing rapid cellular membrane deformation (microfluidics) was used for the delivery of a range of different macromolecules, including anti-tubulin antibodies, into live HeLa cells (Sharei et al., 2013). All these physical delivery methods offer the advantage of direct antibody delivery inside the cytosol with the ability to target other compartments (e.g., the nucleus) upon the attachment of the specific localization signal, as shown for the nuclear delivery of anti-PCNA antibody conjugates with NLS delivered into the cells via electroporation (Freund et al., 2013). Possible limitations of these methods include a lack of cell-specificity, in addition to rather difficult *in vivo*, and difficulties in clinical translation. Examples of different approaches to intracellular physical delivery of antibodies are presented in Table 1.

**Table 1:** Examples of physical delivery of antibodies inside cells.

Delivery approach	Antibody type	Targeted antigen	Reference
Microinjection	monoclonal antibody (mAb) (IgG)	α-tubulin	Lessman et al., 1997
Microinjection	polyclonal antibody labeled with Alexa Fluor 488	inosine-5′-monophosphate dehydrogenase 2	Keppeke et al., 2015
Microinjection	mAbs	N-terminal transactivation region of p53	Gire and Wynford-Thomas, 1998
Microinjection	four different mAbs	α-p21	Ma et al., 1999
Microinjection	polyclonal antibody	fos	Riabowol et al., 1988
Microinjection	polyclonal antibody (IgG)	actin	Scheer et al., 1984
Microinjection into either the nuclei or cytoplasm	NLS-conjugated polyclonal antibody (IgG)	lamin A/C histone-binding site	Dixon et al., 2017
Electroporation	two different mAbs	bovine asparagine synthetase	Chakrabarti et al., 1989
Electroporation	mAbs	proliferating cell nuclear antigen (PCNA) DNA polymerase α, HPV16 E6 oncogene	Freund et al., 2013
Electroporation	mAb and Fab	PCNA DNA polymerase alpha	Desplancq et al., 2016
In situ electroporation	mAbs	TF-1 apoptosis-related gene 19 (TFAR19), or Programmed Cell Death 5(PDCD5)	Rui et al., 2002
Electroporation	scFv-Fc	myosin, tubulin	Marschall et al., 2014
Electroporation	polyclonal antibody	pp60c-src	Marrero et al., 1995
Electroporation	mAb	cyclin D1	Lukas et al., 1994
Electroporation	mAbs and Fabs labeled with Alexa Fluor 488	γH2AX, α-tubulin, heptapeptide repeats of nonphosphorylated C-terminal domain of the largest subunit of RNA Pol II, TATA binding protein (TBP), TBP-associated factor 10	Conic et al., 2018
Microfluidics	mAb labeled with Alexa Fluor 488	tubulin	Sharei et al., 2013
Sonoporation	mAb	E6 HPV 16 oncoprotein	Togtema et al., 2012
Laser-induced cavitation bubbles	mAb labeled with Alexa Fluor 488	α-tubulin	Wu et al., 2015

### Direct Intracellular Expression

In addition to the physical delivery described above, a well-established straightforward method for intracellular antibody delivery is the so-called intrabody technique. An intrabody is an antibody or its derivative produced within the same cell where the antigen is located. Intrabody expression can be achieved by cell transfection with a plasmid or virus carrying the gene encoding the antibody or its fragment. This technology has become more attractive since one of the pioneering proof-of-principle works demonstrating the inhibition of alcohol dehydrogenase I in *Saccharomyces* transfected with cDNA-coding antibody was published in 1988 (Carlson, 1988). The advantages of this approach are the direct expression within the cell and relatively easy direction of intrabody to the desired cell compartment where the specific antigen should be bound (e.g., membrane or secreted protein in the ER or nuclear protein in the nucleus). The latter is usually achieved by the attachment of a targeting sequence. The targeting of intrabodies to be retained in the ER appears to be the most straightforward approach, enabling their maturation and folding in the native environment.

A secretory signal peptide targets intrabody to the ER lumen, and ER retention by KDEL (Lys-Asp-Glu-Leu) (or similar) sequence introduced into the C-terminus of the intrabody traps the intrabody–antigen complex within the ER–Golgi complex (Hammond and Helenius, 1994). These types of intrabodies do not need to be strictly neutralizing to exert their knockdown functions, as the mere act of retention of secretory or membrane proteins within the ER knock down their functionality. In contrast, intrabodies aimed at cytosolic, nuclear, or mitochondrial antigens require much more precise tuning to provide for their appropriate folding within the cytosol (Hammond and Helenius, 1994; Marschall and Dubel, 2016). To overcome this limitation, many different strategies for the generation and/or selection of suitable antibodies for cytosolic expression have been developed. These approaches include: use of single-domain antibody variants [camelid VHH, shark-derived variable new antigen receptors, or variable domain of the heavy chain (VH) or light chain (VL) selected from human antibodies] (Boldicke, 2017); fusion with different protein domains [maltose binding protein (Bach et al., 2001), Fc-domain (Strube and Chen, 2004), proteasome-targeting PEST motif (Joshi et al., 2012), and others (Jurado et al., 2006); grafting of complementary determining regions (Donini et al., 2003); construction and selection of intrabodies lacking S–S bonds (Colby et al., 2004; Cetin et al., 2017); numerous eukaryotic *in vitro* selection-based strategies aimed at isolating a functional and soluble antibody fragment (Guglielmi et al., 2011; Matz et al., 2014; Lee et al., 2016) and even electrostatic manipulation via the introduction of negative charges (Kvam et al., 2010; Liu et al., 2015). In addition to direct interaction with the antigen located in the cytosol, simple fusion with an appropriate signal peptide (Biocca et al., 1991) can redirect intrabodies expressed in the cytosol to bind the antigens within the cell nucleus (Verheesen et al., 2006) or mitochondrion (Biocca et al., 1995).

As phage display technology allows rapid and relatively easy generation of antibodies to almost any antigen of interest (Vaughan et al., 1996; Colwill and Graslund, 2011; Frenzel et al., 2017), intrabodies aimed at many different proteins, including their splice variants and specific post-translational modification sites (Koo et al., 2014; Chirichella et al., 2017), have been developed and shown to be rather efficient *in vitro* and sometimes *in vivo* (Amici et al., 2016). The most widely utilized targets include cancer-linked antigens (Van Impe et al., 2013; Chan et al., 2016; Alirahimi et al., 2017; Amici et al., 2016), neurodegenerative-disease-related antigens (Lynch et al., 2008; Joshi et al., 2012; Butler and Messer, 2011), toxins (Tremblay et al., 2010; Alzogaray et al., 2011), viral infection (e.g., HIV or HCV) – related antigens (Matz et al., 2014; Boons et al., 2014; Ashour et al., 2015; Amici et al., 2016), and miscellaneous antigens, mostly for protein function studies, within the cell (Van Audenhove et al., 2013). The therapeutic potential of intrabodies against cancer (Amici et al., 2016), Huntington’s disease (Southwell et al., 2009), and Alzheimer’s disease (Ryan et al., 2010) has been already demonstrated in mouse models of these diseases. Moreover, in a phase I clinical trial conducted in 2000 (Alvarez et al., 2000), the feasibility of adenoviral-mediated gene therapy using an anti-erbB-2-directed intrabody in the context of human ovarian cancer was shown: the authors reported anti-erbB-2 scFv-encoding gene expression measured by polymerase chain reaction and real-time polymerase chain reaction in most patients with very limited toxicity. However, contrary to the effects demonstrated by an anti-erbB-2-directed intrabody in animal studies (Deshane et al., 1995), no dramatic clinical benefit was observed in this trial, leaving much room for the improvement of this strategy.

Thus, the attractive potential therapeutic use of intrabodies still has unresolved issues; the major issues are finding an efficient and safe DNA transfection method *in vivo* and insufficient tissue specificity. The other issues include the delayed onset of the effect, uncertain duration and level of expression, and whether the transformation is transient or permanent (especially when using such vectors as polyplexes, liposomes, adenoviruses). However, extensive research into the development of promising new nucleic acid delivery systems, including tissue-specific variants (Durymanov et al., 2013; Mann and Kullberg, 2016; Kim et al., 2017) for various gene therapy applications have led to a number of clinical trials and a few already clinically approved approaches (Pearson et al., 2004; Yla-Herttuala, 2012; Buning, 2013; Zhang W.W. et al., 2018) favor the introduction of intrabody-based therapy to the clinic.

### Fusion With Part of Internalizing Autoantibodies Responsible for Their Intrinsic Ability to Enter Cells

Although the intrabody approach relies on straightforward intracellular expression within the same antigen-expressing cells, another strategy is based on the use of the intrinsic ability of several naturally occurring autoantibodies (see Section “Internalization of Antibodies Into Cells Via Normal and Pathological Processes”) to enter the cells. The most straightforward pathway utilizes the recently discovered potential of a subset of cell and nuclear penetrating lupus erythematosus anti-DNA autoantibodies to serve as therapeutic agents targeted toward DNA repair-deficient malignancies (Hansen et al., 2012; Noble et al., 2014, 2015). Specifically, the lupus erythematosus anti-DNA autoantibodies 3E10 (Hansen et al., 2012) and their more potent divalent mutants (Noble et al., 2015), including humanized and re-engineered ones (Rattray et al., 2018), as well as the nucleolytic autoantibody 5C6 (Noble et al., 2014) were shown to bind DNA and either inhibit key steps in DNA repair or damage single-stranded DNA in a manner, making them selectively lethal to cancer cells with defective homology-directed repair of DNA double-strand breaks.

A more flexible and universal approach recruits the fragments responsible for cell entry of those cell penetrating autoantibodies for fusion with antigen-recognizing parts of the antibody of choice. A series of articles (Weisbart et al., 2004b, 2012; Choi et al., 2014; Kim J.S. et al., 2016; Shin et al., 2017) have reported proof-of-principle of the feasibility of this approach to the demonstration of significant antitumor effects *in vivo*. In particular, humanized VL of lupus erythematosus autoantibody m3D8, with its ability to penetrate the cell and localize in the cytosol, has been engineered into different human IgGs to give so-called cytotransmabs capable of targeting an intracellular antigen of choice within the cytosol (Choi et al., 2014). Based on this strategy, the human IgG1 format antibody named iMab RT11-i containing m3D8 humanized VL targeted to the cytosolic activated GTP-bound form of oncogenic Ras mutants has been developed. Decorated with tumor-homing integrin binding RGD10 cyclic peptide, this iMab inhibited the growth of oncogenic Ras-mutated tumor xenografts in mice, but not wild-type Ras-harboring tumors (Shin et al., 2017), demonstrating the feasibility and potential of this approach for the cytosolic delivery of antibodies. Nuclear delivery of antibodies has been demonstrated by using a bivalent scFv derivative of cell and nuclear penetrating autoantibody 3E10 fused to an anti-MDM2 antibody, which impaired the growth of melanoma cells and melanoma xenografts sensitive to MDM2 inhibition (Weisbart et al., 2012).

### Protein-Transduction Domains or Their Mimics

Contrary to the limited number of studies utilizing the part of internalizing autoantibodies responsible for their intrinsic ability to enter the cells, a somewhat similar approach relying on fusion to protein transduction domains, or CPPs, is a widely utilized technique to shuttle various cargos (proteins, plasmid DNA, RNA, oligonucleotides, liposomes, imaging agents, and anti-cancer drugs) into living cells. Since the discovery of the first and prototypical HIV 1 trans-activating (TAT) protein, capable of cell membrane penetration (Green and Loewenstein, 1988; Frankel and Pabo, 1988) in 1988, a wide variety of CPPs and some of their mimics was derived and recruited for the intracellular delivery of various agents for research studies or potential therapeutic applications (Kristensen et al., 2016; Bolhassani et al., 2017; Borrelli et al., 2018), with a few reaching clinical trials to date (NCT01975116, NCT00914914). Unsurprisingly, this promising strategy has also been attempted to enhance intracellular antibody delivery, which has led to cell penetrating TransMabs (Muller et al., 2005) or transbodies. An early study (Anderson et al., 1993), revealing enhanced cell retention and internalization of Fab fragments decorated with a TAT protein-derived peptide, was followed more than a decade later by a set of articles demonstrating that antibody-based fusion proteins containing a short membrane transport-facilitating peptide were transported into the cells and bound to intracellular structures (Zhao et al., 2001) and that anti-caspase-3 antibody based TransMabs were able to inhibit apoptosis (Zhao et al., 2003) in cells. Subsequently, this approach gained popularity and the number and use of CPP-conjugated whole antibodies, and their derivatives, started to increase (Mie et al., 2003; Cohen-Saidon et al., 2003; Ohara-Imaizumi et al., 2004; Shin et al., 2005; Chen and Erlanger, 2006; Theisen et al., 2006; Hu et al., 2007; Avignolo et al., 2008); see Table 2 for examples. Recently, a transbody based on mAbs against human HBcAg coupled with the TAT protein transduction domain was demonstrated to be effective *in vivo* (Li et al., 2017).

**Table 2:** Examples of intracellular delivery of CPP-fused antibodies.

CPP or their mimics used for fusion	Antibody type	Targeted antigen	Reference
TAT peptide	Fab	melanoma-associated antigen, pan-carcinoma antigen	Anderson et al., 1993
Membrane transport sequence	IgG	human B-cell lymphoma, mouse B-cell tumor	Zhao et al., 2001
Poly-L-arginine (average molecular weight 10,750, ca. 68 residues)	mAb	HIV-1 Gag, fullerene and others	Chen and Erlanger, 2002
TAT peptide	scFv	Bcl-2	Cohen-Saidon et al., 2003
TAT fusion protein A	IgG	–	Mie et al., 2003
TAT peptide	mAb	syntaxin 1	Ohara-Imaizumi et al., 2004
Membrane-translocating sequence (MTS) from Kaposi fibroblast growth factor	scFv	Akt	Shin et al., 2005
Polyarginine 68R	mAb	cyclin D1	Chen and Erlanger, 2006
TAT peptide	scFv	HIV-1 TAT-protein	Theisen et al., 2006
TAT peptide	IgG	p21 WAF-1/Cip-1	Hu et al., 2007
Antennapedia protein transduction domain	scFv	c-Myc	Avignolo et al., 2008
Penetratin (PEN) of the Drosophila homeodomain	scFv	M1 matrix protein of influenza A virus	Poungpair et al., 2010
BR2 (17aa peptide)	scFv	K-RAS	Lim et al., 2013
TAT peptide	Fab	HIV-1 protein Rev	Zhuang et al., 2014
3R-Based lipophilic protein A-modified polymer	mAb	nuclear pore complex	Itakura et al., 2015
Anthrax toxin protective antigen	affibody, DARPin, monobody, protein GB1	Src homology 2 domain of the oncoprotein Bcr-Abl	Liao et al., 2014
Fc-binding peptide (FcBP)-TAT conjugate	IgG	–	Kang et al., 2015
Eight different CPPs	IgG	HIV-1 p24 protein	Ali et al., 2016
GET: membrane-docking peptide to heparan sulfate glycosaminoglycans (GAGs) fused with a PTD (peptide P21 and 8R)	IgG	–	Dixon et al., 2016
Nona-arginine (R9)	scFv	(NS3/4A) of HCV	Jittavisutthikul et al., 2016
Nona-arginine (R9)	scFv	Ebolavirus VP40	Teimoori et al., 2016
Protein transduction domain mimics MePh13-b-dG5 (P13D5)	IgG	phosphorylated protein kinase C𝜃 (Thr538)	Ozay et al., 2016
Mutated several amino acid residues on the surface of the proteins to basic residues, resulting in net positive charges of +14 and +15	nanobodies	green fluorescent protein (GFP), HER2, and β-lactamase	Bruce et al., 2016
Cyclic R10 (cR10) peptide	nanobodies	GFP	Herce et al., 2017
Nona-arginine (R9)	ScFv	(NS5A) of HCV	Glab-Ampai et al., 2017
TAT peptide	mAb	HBcAg	Li et al., 2017
Co-administration with endosomolytic peptides derived from the cationic and membrane-lytic spider venom peptide M-lycotoxin	IgG	–	Akishiba et al., 2017
TAT peptide	IgG1 mAb	Hepatitis B virus X protein	Zhang J.F. et al., 2018
Nona-arginine (R9)	HuscFvs	interferon inhibitory domain of the VP35 protein, a multifunctional virulence factor of Ebola virus	Seesuay et al., 2018
Cell-penetrating poly(disulfide)s	IgG labeled with Cy5	–	Qian et al., 2018

However, there are two main limitations to be addressed when using this strategy. The first stems from the pool of data that demonstrate entrapment of the internalized CPPs (Richard et al., 2003) and CPP-conjugates inside endocytic vesicles (Fischer et al., 2006; Benavent Acero et al., 2014; Marschall et al., 2014), which has raised the question as to whether CPPs and CPP-conjugated macromolecules, including transbodies, are able to be released from the endosomes efficiently, making efficient endosomal escape one of the limitations of this approach (El-Sayed et al., 2009). The insertion of an additional endosomolytic moiety was suggested to improve the situation (Rinne et al., 2007; Liou et al., 2012). A recent study reported an efficient endosomal escape of an antibody co-delivered with an endosomolytic peptide derived from the cationic and membrane-lytic spider venom peptide M-lycotoxin (Akishiba et al., 2017). An approach utilizing light-controlled endosomal photosensitizer-driven escape of the construct based on an anti-Ki67 mAb decorated with CPP Pep-1 co-delivered with photosensitizer was revealed as an effective strategy to facilitate the endosomal escape of antibodies (Wang et al., 2015).

Another limitation of CPP-based technology, including transbodies, may be the absence of tissue and cellular specificity. Although, in the case of transbodies, cell-specific action can be reached on the antigen level, the problem of unnecessary unspecific delivery to cells remains, but may be solved by using some cell-homing CPPs (Svensen et al., 2012) or the addition of a cell-targeting ligand to a transbody. For example, a fusion of anti-mutated K-ras scFv and cancer-cell specific CPP BR2 demonstrated significant and cancer-cell-selective effects *in vitro* (Lim et al., 2013).

### Nanocarriers

Nanocarrier-driven targeted intracellular delivery of different payloads is a widespread strategy that has the following advantages: allows systemic delivery of a relatively large amount of cargo with reduced cargo-related side effects; lowers off-target organ toxicity; enables the possibility of sustained release; and shields the cargo from premature enzyme degradation (Sousa et al., 2017). Moreover, nanocarriers permit relatively easy decoration with functional (e.g., cell-targeting) moieties, which, in contrast to direct antibody modification, cannot hamper their reactivity. Thermosensitive (Fathi et al., 2018), enzyme-sensitive (Aluri and Jayakannan, 2017), pH-sensitive (Braunova et al., 2017), light-responsive (Kim H. et al., 2016), and combo-stimuli-responsive (Kashyap et al., 2016) nanocarriers can be easily designed to provide a controlled payload release in response to thermal (body, increased tumor temperature, or localized heating), specific enzymatic (e.g., tumor microenvironment or intracellular enzymes) and specific pH microenvironments (e.g., acidic tumor microenvironment or acidifying endocytic vesicles) as well as to external light exposure or any combination of these.

Various types of nanocarriers (see Table 3 for examples), including inorganic nanoparticles (Chiu et al., 2016; Song et al., 2016; Amornwachirabodee et al., 2018), polymersomes (Liu et al., 2010; Tian et al., 2015), liposome-based nanocarriers (Chatin et al., 2015), as well as modified viral (Kondo et al., 2008) and virus-like particles (Abraham et al., 2016) have all been recruited for antibody intracellular delivery with reasonable success.

**Table 3:** Examples of intracellular delivery of antibodies by nanocarriers.

Nanocarrier	Antibody type	Targeted antigen	Reference
IgG-lipopolyamine dioctadecylglycylspermine complexes	IgG	β-actin, α-tubulin	Dalkara et al., 2004
Cationic lipid-based complexes	mAb	HPV16 E6 oncoprotein	Courtete et al., 2007
TAT-HA2 decorated gold nanoparticles	glycosylated mAbs	actin	Kumar et al., 2007, 2008
Protein A Z-subdomain dimer fused with nucleocapsid protein incorporated into hemagglutinating virus of Japan envelope	IgG	α-tubulin nuclear pore complex	Kondo et al., 2008
Gold-coated iron oxide nanoparticles or quantum dots decorated with Streptococcal bacterial protein G fused with TAT	mAb	mitochondria	Lim et al., 2009
Polymersomes	IgG	–	Liu et al., 2010
Polymersomes	IgG	NF-κB and γ-tubulin, actin, Golgi protein	Canton et al., 2013
Self-associated MAb nanoparticles	mAb bevacizumab	VEGF	Srinivasan et al., 2013
Self-assembling pyridylthiourea-modified polyethylenimine nanoparticles	mAbs modified with NLS	HPV-16 viral E6 oncoprotein, threonine-927 phosphorylation site of the EG5 kinesin spindle protein	Postupalenko et al., 2014
Liposomes based on guanidinium-cholesterol cationic lipid BGTC combined to the colipid (DOPE)	mAb	cytokeratin8	Chatin et al., 2015
Polymersomes targeted to low density lipoprotein receptor-related protein 1 receptor	IgG	–	Tian et al., 2015
Virus-like particles decorated with IgG binding moiety	IgG	abrin, anti-α-tubulin, HER2	Abraham et al., 2016
Mesoporous silica nanoparticles	chromobody: fluorescent nanobodies conjugated with fluorescent proteins or organic dyes	GFP	Chiu et al., 2016
Polyion complex micelles	IgG Fc	nuclear pore complex	Kim A. et al., 2016
Nanoparticles based on nanobodies with sequence-defined oligoaminoamides decorated with folic acid	nanobodies	GFP	Roder et al., 2017
Calcium phosphate biomineralization	mAbs	Dengue virus surface envelope glycoprotein, Hemagglutinin (HA) protein of Influenza A virus	Song et al., 2016
Erythrocyte membrane coated self-assembling nanoparticles	mAb	hTERT	Gao et al., 2017
α-helical peptide Hex nanocarrier decorated with Aurein	IgG	anti-β-tubulin or anti-nuclear pore complex	Lim et al., 2017
Oxidized carbon black particles	mAb	Dengue virus	Amornwachirabodee et al., 2018

#### Lipid-Based Nanocarriers

Lipid-based nanoparticles are extensively used for the intracellular delivery of therapeutic proteins, including antibodies. Several cationic-lipid based nanocarriers (Dalkara et al., 2004; Courtete et al., 2007) have been proposed for the intracellular delivery of antibodies. For example, Courtete et al demonstrated that a lipid reagent for siRNA transfection could be successfully adjusted to give efficient anti-HPV16 E6 antibody delivery and resulted in the downregulation of this oncoprotein activity *in vivo* (Courtete et al., 2007).

#### Polymer Based Nanocarriers

Polymer based nanoparticles are another widely used approach for intracellular protein delivery. Several years ago, polymer-based liposomes, called polymersomes (Discher et al., 1999) were shown to be effective for the cytosolic delivery of antibodies aimed at various intracellular antigens inside the cells, resulting in the respective epitope binding and subsequent biological effect (Canton et al., 2013) in different cell types, as no cell-specific moiety was required in this proof-of-principle study. A few years later, a sophisticated polymersome-based approach for cell-specific antibody delivery across the blood–brain barrier was developed (Tian et al., 2015). Polymersomes were decorated with Angiopep-2 peptide targeting low density lipoprotein receptor-related protein 1 receptor to yield dual functionality, as this receptor is associated with both endothelial transcytosis and endocytosis into the cells of the central nervous system. As a result, Angiopep-2-decorated polymersomes delivered IgGs into the central nervous system cell *in vitro*, efficiently crossed the blood–brain barrier, and co-localized with astrocytes, neurons, and glial cells *in vivo* (Tian et al., 2015); thus, this approach warrants further development.

#### Inorganic Nanocarriers

Inorganic nanoparticles composed of several inorganic materials have been used for the intracellular delivery of antibodies. For example, mesoporous silica nanoparticles functionalized with nitrilotriacetic acid–metal ion complexes for pH-sensitive binding and release of the delivered His6-tagged chromobodies (anti-GFP fluorophore labeled nanobodies) exhibited the ability to deliver these chromobodies inside the cells (Chiu et al., 2016). A noticeable release of chromobodies to cytosol required the use of endosomal release triggers, of which chloroquine and dimethyl sulfoxide were the most effective ones, which may be unsuitable for practical use. Another group developed mAbs to anti-influenza A and anti-Dengue viral proteins biomineralized with calcium phosphate, yielding sphere-like 130–150 nm nanoparticles, which, in contrast with native unmodified mAbs were able to enter the cells and co-localize with their antigens within the endosomal compartment. Moreover, the biomineralized mAb led to a significant reduction in the mortality of the infected mice compared with the corresponding naked mAb (Song et al., 2016). Both these nanoparticle-based delivery systems possessed difficulties in endosomal escape of the delivered antibody that necessitated the use of endosomal release triggers or were limited to antigens that are known to localize within the endosomes. An easy way to reach the cytosol is to enter the cell by a non-endocytosis-involving pathway, as recently demonstrated for oxidized carbon black nanoparticles, which are supposed to cause temporary disruption of the lipid bilayer and thus provide antibody delivery inside the cells via an electroporation-like mechanism (Amornwachirabodee et al., 2018). Another solution to facilitate the delivery of antibody-bearing nanoparticles into the cytosol been proposed by Lim et al. (2009), who decorated gold-coated iron oxide nanoparticles with IgG-binding streptococcal bacterial protein G fused with TAT peptide for endosomal escape. The resulting functionalized magnetic nanoparticles loaded with anti-mitochondria mAb were able to enter the cell and reach mitochondria within HeLa cells (Lim et al., 2009). However, the efficiency of TAT peptide-mediated endosomal escape is supposed to be rather low (Richard et al., 2005); thus, in order to improve endosomal release, another group developed an approach that recruited gold nanoparticles decorated with TAT peptide fused with pH-sensitive influenza virus hemagglutinin protein HA2; in this, the former moiety was responsible for cell-entry and the latter for the introduction of efficient endosomal release of the delivered anti-actin antibody (Kumar et al., 2007).

The main limitation of all these approaches is their non-specific action; however, this may be solved by additional inclusion of nanoparticles with a cell-homing moiety.

#### Viral and Virus-Like Nanocarriers

Being extensively used for gene delivery, viral-based vehicles have been recruited successfully for intracellular antibody delivery. Efficient entrapment of the antibody within these viral-based nanoparticles is often provided by the inclusion of *Staphylococcus aureus* protein A or its sub-domains capable of IgG binding (Kondo et al., 2008; Park et al., 2009; Abraham et al., 2016). A decade ago, a system based on a protein A Z-subdomain dimer fused with nucleocapsid protein incorporated into the envelope of hemagglutinating virus of Japan revealed the ability to efficiently incorporate IgGs and target them to the respective intracellular antigens (nuclear pore complex and α-tubulin) (Kondo et al., 2008). Owing to the possible toxic and immunogenic issues connected with viral vectors of animal origin, intracellular antibody-delivering plant virus-like nanoparticles, which are considered to be nonpathogenic in humans, were recently developed (Abraham et al., 2016). Based on Sesbania mosaic virus coat protein modified with IgG binding protein A B-domain self-assembling virus-like particles, this platform for intracellular delivery of antibodies proved to be effective for intracellular delivery of three different mAbs, which exerted their biological effects owing to respective epitope binding (Abraham et al., 2016). Generally, owing to the high efficiency of antibody delivery across the membrane, combined with the high loading efficiency compared with most nanovehicles (e.g., inorganic nanoparticles or polymersomes), viral-based particle approaches will encourage new horizons of study in this field, especially if an additional ability to target only the specified cells can be introduced into this type of nanoparticle.

#### Other Types of Nanocarriers

A few self-assembled protein nanocarriers (Lim et al., 2017), polyion complex micelles (Kim A. et al., 2016), and pharmacokinetic improving erythrocyte membrane-coated mAb nanoparticles (Gao et al., 2017) have been developed, with some exhibiting encouraging results. For example, a self-assembled protein nanocarrier based on an α-helical peptide self-assembling into a hexameric coiled-coil bundle fused with an Fc-binding Protein A fragment was recently developed. It was further decorated with an endosomolytic Aurein 1.2 sequence. This nanocarrier enabled a high antibody loading and efficient delivery into the cytosol (Lim et al., 2017). The authors proposed possible future modifications of this nanocarrier with cell specific targeting ligands to dramatically increase the attractiveness of this already promising nanovehicle.

### Strategies for Intracellular Targeting of Antibodies, Their Mimics, and Derivatives: Summary of the Limitations and Prospects

Targeted intracellular delivery of antibodies can be aimed at the antigens localized in various intracellular compartments, predominantly the ER, cytosol, and nucleus. Examples of efficient antibody intracellular delivery approaches aimed at specified intracellular compartments are presented in Table 4, and the advantages and limitations of different approaches are summarized in Table 5. The ER is very easily targeted by intrabodies, as natural antibodies already contain a signal for secretion; thus, only the additional ER retention motif (KDEL, usually) should be encoded within the antibody gene to achieve delivery into the cell, where an intrabody is expressed. Cytosolic and nuclear antigens require more effort to achieve intrabody targeting, but are still vastly applicable. The main problem with regard to the use of intrabodies is the requirement of a safe, efficient, and cell-specific gene delivery method for their *in vivo* use. The targeted delivery of the antibodies themselves inside the cell from the outside seems to lack many of the safety parameters associated with gene delivery, but narrows the variety of intracellular compartments that have been reached to date by the delivered antibody; to the best of our knowledge, no approaches for targeting the ER by an externally delivered antibody have yet been published. An efficient cell-specific targeted delivery of antibody for the intracellular antigen of interest from the cell outside requires a stepwise delivery, starting from the specific recognition of the target cells, followed by effective internalization of the construct, with the subsequent endosomal escape to the cytosol and – if necessary – further transport to the required cell compartment (e.g., the nucleus or mitochondria). Unfortunately, just a few published delivery strategies integrate all these crucial steps together in one construct. For example, a recent approach for the construction of modular transport systems, including DARPins, for cell specific receptor recognition, bacterial toxin-derived component for endosomal escape, and a different DARPin for intracellular antigen recognition (Verdurmen et al., 2015) was published. This system was demonstrated to deliver DARPins into the cytosol efficiently and cell-specifically. When an antigen of interest is localized within another compartment (e.g., the nucleus or mitochondria), additional signal peptides for specific compartment delivery are required. The delivery system that can serve an appropriate candidate to accomplish this step-by-step targeted cell-specific delivery of antibody (or its derivative/mimic) into the designated intracellular compartment (e.g., the nucleus) is the modular nanotransporters platform (Sobolev, 2009; Sobolev et al., 2016). Modular nanotransporters are recombinant polypeptide-based delivery vehicles, consisting of a ligand module for cell-specific recognition and subsequent internalization, the translocation domain of Diphtheria toxin as an endosomolytic module for successful endosomal escape, and the optimized SV-40 large T-antigen nuclear localization sequence for nuclear import. The feasibility of this platform has already been demonstrated *in vitro* and *in vivo* for the delivery of locally acting anti-cancer drugs, such as photosensitizers (Slastnikova et al., 2012b,c), several types of radionuclides [α-emitters (Rosenkranz et al., 2008), and Auger electron emitters (Slastnikova et al., 2012a, 2017b; Koumarianou et al., 2014)] into various types of cancer cells. The cancer cell specificity was easily modulated by the choice of appropriate ligand module [e.g., epidermal growth factor (Gilyazova et al., 2006), α-melanocyte stimulating hormone (Rosenkranz et al., 2003), and folic acid (Rosenkranz et al., 2017; Slastnikova et al., 2017a)] for the characteristic internalizable receptor overexpressed on the target cells. Recently, the principal feasibility of the construction of modular nanotransporters bearing anti-cMyc scFv (as an example) was demonstrated (Ulasov et al., 2018), which may be a promising new step toward the efficient targeted delivery of antibodies inside the target cells.

**Table 4:** Examples of efficient intracellular delivery of antibodies aimed at various intracellular compartments.

Intracellular compartment(s) to which antibodies or mimics were successfully delivered	Level of action (*in vitro* or *in vivo*)	Type of delivery (carrier if applicable)	Antibody type	Targeted antigen	Reference
Cytosol	*in vitro*	TAT-HA2 decorated gold nanoparticles	glycosylated mAbs	actin	Kumar et al., 2007, 2008
Cytosol	*in vitro* and *in vivo*	humanized VL of lupus erythematosus autoantibody m3D8 engineered into human IgG decorated with tumor homing RGD10 cyclic peptide	IgG	cytosolic activated GTP-bound form of oncogenic Ras mutants	Shin et al., 2017
Cytosol	*in vitro*	modular transport systems including DARPin for cell specific receptor recognition and bacterial toxin-derived component for endosomal escape and a different DARPin for intracellular antigen recognition	designed ankyrin repeat protein (DARPin)	mainly none (model cargo DARPins)	Verdurmen et al., 2015
Endoplasmic reticulum (ER)	*in vitro*	intrabody technology: diethylaminoethyl-dextran based transfection	scFv fused with ER retention KDEL signal	human herpesvirus 8 interleukin-6	Kovaleva et al., 2006
Mitochondria, nucleus, or cytosol	*in vitro* in cell lines (all localizations) and in Xenopus oocytes (mitochondria)	intrabody technology: transient DNA transfection of cells, mRNA microinjection to Xenopus oocytes	scFv fused with nuclear or mitochondrial localization signals	p21ras, nerve growth factor	Biocca et al., 1995
Nucleus	*in vitro*	self-assembling pyridylthiourea-modified polyethylenimine nanoparticles	mAbs modified with NLS	HPV-16 viral E6 oncoprotein	Postupalenko et al., 2014
Nucleus	*in vitro* and *in vivo*	scFv of cell and nuclear penetrating autoantibody 3E10 fused to anti-MDM2 antibody	mAb	MDM2	Weisbart et al., 2012
Nucleus	*in vitro*	electroporation	mAbs	proliferating cell nuclear antigen (PCNA) DNA polymerase α, HPV16 E6 oncogene	Freund et al., 2013
Nucleus	*in vitro*	electroporation	mAbs and Fabs labeled with Alexa Fluor 488	γH2AX, α-tubulin, heptapeptide repeats of nonphosphorylated C-terminal domain of the largest subunit of RNA Pol II, TATA binding protein (TBP), TBP-associated factor 10	Conic et al., 2018
Nucleus	*in vitro*	microinjection into either the nuclei or the cytoplasm	NLS-conjugated polyclonal antibody (IgG)	lamin A/C histone-binding site	Dixon et al., 2017

**Table 5:** Advantages and limitations of different approaches used for the intracellular delivery of antibodies.

Delivery approach	Advantages	Current limitations
Direct physical delivery	delivers antibodies directly inside the cytosol; the ability to target other compartments upon attachment of the specific localization signal	usually lacks cell-specificity; rather difficult *in vivo* and clinical translation owing to *in vivo* safety and efficiency issues
Direct intracellular expression (intrabody approach)	direct expression within the cell; relatively easy direction of intrabody to the desired cell compartment where the specific antigen should be bound	rather difficult *in vivo* and clinical translation owing to DNA transfection *in vivo* safety and efficiency issues; insufficient tissue specificity; delayed onset of the effect; uncertainties with duration and level of expression; uncertainty whether the transformation is transient or permanent
Fusion with part of internalizing autoantibodies responsible for their intrinsic ability to enter cells	relies on intrinsic abilities of autoantibodies to enter the cell; can be easily re-engineered to obtain more potent derivatives; can be additionally decorated with tissue targeting moieties	mechanism of cell entry and endosomal escape not clearly understood; endosomal escape efficiency issues lack of tissue and cellular specificity; additional decoration can hamper antibody reactivity
Fusion with protein-transduction domains or their mimics	can be additionally decorated with tissue targeting and endosomal escape moieties	questionable efficiency of endosomal escape; generally lacks tissue and cellular specificity; additional decoration can hamper antibody reactivity possible toxicity issues
Nanocarriers	generally high loading efficiency; tunable properties of the carrier, allow rather easy decoration with functional (e.g., cell-targeting) moieties, which in contrast to direct antibody modification cannot hamper its reactivity possible sustained release functionality; possible adjustments of the pharmacokinetic profile	for the majority of nanocarrier types, the problem of efficient endosomal escape still needs to be solved; production can be highly tedious and expensive; immunogenicity issues due to relatively large size of nanoparticles

### Directed Subcellular Relocation of Target Molecules

Another theme that emerges from the intracellular antibody delivery concept is the ability to relocate protein-of-interest within the cells or deplete it through a cellular protein elimination system. Protein relocation is an approach to interfere with target protein function through trapping it with binder molecule and thereby preventing the target protein transport to a defined cellular compartment. The feasibility of such strategy was demonstrated with intracellular antibodies, which inhibited heterochromatin protein 1β traffic to the nucleus (Cardinale et al., 2015), resulting in altered nuclear morphology and apoptosis. Another group reported intracellular antibody binding to Sec61 and prevention of its transport from the ER toward endosomes (Zehner et al., 2015). To interfere with the target protein function, the intracellular antibody must be: (1) delivered into cells at a stoichiometric concentration; (2) bound to the target in a way that blocks its function, and (3) ensured for high affinity binding, otherwise the equilibrium will shift toward target escape from antibody complex before reaching desirable effect. These are strict conditions that limit the possible applications of intracellular antibodies against relatively low-abundant targets and/or molecules with a very high affinity toward the target.

Notwithstanding the elimination of the target, once bound to the antibody, it could circumvent this equilibrium conundrum. For specific degradation of proteins, cells contain proteasome systems, which are highly efficient and elaborate machinery for the regulation of protein turnover and protection from misfolded and damaged proteins (Bhattacharyya et al., 2014). A ubiquitin-dependent system is the major pathway for specific protein degradation, for which protein ubiquitination is a prerequisite step (Hershko and Ciechanover, 1998). Ubiquitination is a multistep enzymatic process, that involves three types of enzyme: E1 ubiquitin activating enzyme; E2 ubiquitin conjugating enzyme; and E3 ubiquitin protein ligase (Komander and Rape, 2012). The substrate specificity is conferred by hundreds of E3 ubiquitin ligases (Senft et al., 2018), belonging to HECT, RING, and U-box families, according to their mode of action. RING and U-box E3 ubiquitin ligases function as scaffolds for E2 ubiquitin conjugating enzymes, whereas HECT proteins form a thiol-ester bond with ubiquitin before transferring it to the substrates (Pickart, 2001). Harnessing an endogenous protein control system is a viable approach to downregulate the target proteins (Schrader et al., 2009). For this purpose, the binder moiety should be fused with a module targeting the protein to the proteasomes. Several examples of such reprogrammed E3 ubiquitin ligases have been described, based on multi-subunit RING E3 enzymes, targeting β-catenin (Su et al., 2003; Liu et al., 2004) or mono-molecule U-box protein CHIP E3 ligase, degrading KRAS (Ma et al., 2013; Pan et al., 2016), c-Myc (Hatakeyama et al., 2005), epidermal growth factor receptor mutants (Chung et al., 2016). Fusion with CHIP has several advantages over RING E3 ligases, such as broad substrate diversity and a lack of dependence on other subunits (Portnoff et al., 2014). By virtue of the catalytic nature of the ubiquitin system, enabling multiple rounds of activity, depletion of abundant targets, even exceeding the amount of delivered antibody may be possible.

## Conclusion

Three decades of progress in strategies for efficient targeted intracellular delivery of antibodies has led to the development of various approaches, from direct physical methods and gene delivery to sophisticated synthetic delivery vehicles. Although widely utilized for intracellular protein function studies, the therapeutic applications of this promising precise approach remains in the *in vitro* stages and rarely progresses to *in vivo* studies; thus, there is much room for improvement. We hope that both the elucidation of normal behavior of antibodies within cells, including their relocation and degradation pathways, as well as the development of specific, effective, and clinically applicable systems of targeted intracellular protein delivery will allow this approach to be used as a clinical therapy in future.

## Author Contributions

TS, AU analyzed the literature, wrote the article, and designed the figures and tables in the article. AR and AS analyzed the literature, wrote the article, and critically reviewed the article.

## Conflict of Interest Statement

The authors declare that the research was conducted in the absence of any commercial or financial relationships that could be construed as a potential conflict of interest.

## Abbreviations

CPP: cell penetrating peptide
DARPin: designed ankyrin repeat protein
ER: endoplasmic reticulum
Fab: antigen-binding fragment
GFP: green fluorescent protein
HCV: human hepatitis C virus
HIV: human immunodeficiency virus
HPV: human papilloma virus
IgG: immunoglobulin G
mAb: monoclonal antibody
NLS: nuclear localization signal
PCNA: proliferating cell nuclear antigen
PPI: protein–protein interactions
ScFv: single-chain variable fragments
TAT protein: HIV 1 trans-activating protein
VL: variable domain light chain.
